# Inflammatory dysregulation of monocytes in pediatric patients with obsessive-compulsive disorder

**DOI:** 10.1186/s12974-017-1042-z

**Published:** 2017-12-28

**Authors:** Natalia Rodríguez, Astrid Morer, E. Azucena González-Navarro, Carles Serra-Pages, Daniel Boloc, Teresa Torres, Susana García-Cerro, Sergi Mas, Patricia Gassó, Luisa Lázaro

**Affiliations:** 1 Department of Basic Clinical Practice, University of Barcelona, Barcelona, Spain; 2 Department of Child and Adolescent Psychiatry and Psychology, Institute of Neurosciences, Hospital Clinic de Barcelona, Barcelona, Spain; 3 Immunology Service, Hospital Clinic de Barcelona, Barcelona, Spain; 4 Department of Medicine, University of Barcelona, Barcelona, Spain; 5 Department of Biomedicine, University of Barcelona, Barcelona, Spain; 6 Institut d’Investigacions Biomèdiques August Pi i Sunyer (IDIBAPS), Barcelona, Spain; 7 Centro de Investigación Biomédica en Red de Salud Mental (CIBERSAM), Madrid, Spain

**Keywords:** Obsessive-compulsive disorder, Children, Immune system, Microglia, Monocytes, Cytokines, Inflammation

## Abstract

**Background:**

Although the exact etiology of obsessive-compulsive disorder (OCD) is unknown, there is growing evidence of a role for immune dysregulation in the pathophysiology of the disease, especially in the innate immune system including the microglia. To test this hypothesis, we studied inflammatory markers in monocytes from pediatric patients with OCD and from healthy controls.

**Methods:**

We determined the percentages of total monocytes, CD16+ monocytes, and classical (CD14^high^CD16−), intermediate (CD14^high^CD16^low^), and non-classical (CD14^low^CD16^high^) monocyte subsets in 102 patients with early-onset OCD and in 47 healthy controls. Moreover, proinflammatory cytokine production (GM-CSF, IL-1β, IL-6, IL-8, and TNF-α) was measured by multiplex Luminex analysis in isolated monocyte cultures, in basal conditions, after exposure to lipopolysaccharide (LPS) to stimulate immune response or after exposure to LPS and the immunosuppressant dexamethasone.

**Results:**

OCD patients had significantly higher percentages of total monocytes and CD16+ monocytes than healthy controls, mainly due to an increase in the intermediate subset but also in the non-classical monocytes. Monocytes from OCD patients released higher amounts of GM-CSF, IL-1β, IL-6, IL-8, and TNF-α than healthy controls after exposure to LPS. However, there were no significant differences in basal cytokine production or the sensitivity of monocytes to dexamethasone treatment between both groups. Based on monocyte subset distribution and cytokine production after LPS stimulation, patients receiving psychoactive medications seem to have an intermediate inflammatory profile, that is, lower than non-medicated OCD individuals and higher than healthy controls.

**Conclusions:**

These results strongly support the involvement of an enhanced proinflammatory innate immune response in the etiopathogenesis of early-onset OCD.

**Electronic supplementary material:**

The online version of this article (10.1186/s12974-017-1042-z) contains supplementary material, which is available to authorized users.

## Background

Obsessive-compulsive disorder (OCD) is a neuropsychiatric disease characterized by recurrent obsessions and/or compulsions that are distressing, time-consuming, or significantly impairing [1]. It is the fourth most common psychiatric illness, with a lifetime prevalence of 1–3% [2]. OCD presents a bimodal distribution for age at onset with a peak at 12–14 years (early-onset) and another at 20–22 years (late-onset). Indeed, in 30–50% of patients, obsessive-compulsive symptoms start to develop in childhood [3, 4]. It has been proposed that childhood-onset OCD may be a distinct form of the disorder with different etiopathogenic mechanisms [4, 5].

Evidence from different lines of research suggest a possible role of immune dysregulation in the pathophysiology of OCD [6–8]. The hypothesis of immune dysregulation in OCD was originally based on the association found between streptococcal infections and the abrupt onset of obsessive-compulsive symptoms. In this regard, Swedo et al. reported an increased frequency of obsessive-compulsive symptoms and OCD in children with Sydenham’s chorea (SC), a delayed neurological complication following streptococcal infections [9]. The syndrome was named Pediatric Autoimmune Neuropsychiatric Disorders Associated with Streptococcal Infections (PANDAS), a subset of childhood tic disorder and/or OCD which may be causally related to group Aβ-hemolytic streptococcal infection [10, 11].These findings were confirmed in subsequent studies [12, 13] and were also extended to patients with a past streptococcal infection without SC symptoms [7, 14]. Although there is some controversy, it has been hypothesized that this phenomenon may be due to the production of antibodies against basal ganglia structures [6, 7, 11]. In addition to streptococcus infection, other infectious diseases including *Borrelia burgdorferi*, mycoplasma, *Toxoplasma gondii*, or Borna disease virus have been associated with OCD [7, 15]. As different etiologic factors, both infectious and non-infectious, may be involved, the term PANDAS evolved later to a more wide spectrum of acute neuropsychiatric syndrome named PANS (pediatric acute-onset neuropsychiatric syndrome) [15]. These findings have led to increased interest in the involvement of immunological mechanisms in the etiopathogenesis of OCD, regardless of whether it fulfills the diagnostic criteria for PANDAS or not. Some studies have suggested a dysregulation of the immune function in OCD based on alterations in innate and adaptive immune-related parameters such as proinflammatory cytokine levels [16–18], antineural antibodies [8, 19, 20], or hypothalamus-pituitary-adrenal axis dysregulation [21, 22]. However, the results to date are inconclusive.

Several lines of evidence suggest that dysfunction of innate immunity, including the microglia, the brain’s resident immune cells derived from the monocyte lineage, may occur in a number of neuropsychiatric conditions [23–26]. The innate immune system has a pivotal role in initiating, directing, and prolonging the immune response. The main immune cells involved in innate response are monocytes, which circulate in the blood, promote rapid responses, and orchestrate inflammation through the release of proinflammatory cytokines [27]. In addition, this system participates in neuroprotection and neurodevelopment [28]. Human blood monocytes are a heterogeneous population that can be segregated into three functionally different subsets based on their expression of CD14 and CD16: classical (CD14^high^CD16−), intermediate (CD14^high^CD16^low^), and non-classical (CD14^low^CD16^high^) monocytes [29]. CD16+ monocytes (including intermediate and non-classical monocytes [30]) and specifically intermediate monocytes have been termed “proinflammatory” based on their higher production of inflammatory cytokines such as interleukin 6 (IL-6), IL-1β, and tumor necrosis factor alpha (TNF-α) and because of the expansion of these cells in infections and inflammatory diseases [30, 31]. However, the particular state of these cells in OCD is not well known.

Hence, to improve our understanding of the potential role of the innate immune dysregulation in early-onset OCD, we aimed (1) to characterize the monocyte subsets in pediatric patients with OCD and healthy controls, (2) to examine the functionality of cultured monocytes by measuring proinflammatory cytokine and chemokine production after exposure to immune regulators in both OCD and controls, and (3) to explore the relationship of these immune parameters with clinical characteristics of OCD patients.

## Methods

### Subjects

One hundred and two children and adolescents aged between 8 and 19 years with a current diagnosis of obsessive-compulsive disorder (OCD) according to DSM-IV [32] criteria were recruited from the Department of Child and Adolescent Psychiatry and Psychology at the Hospital Clínic in Barcelona. The Spanish version [33] of the semi-structured diagnostic interview K-SADS-PL (Schedule for Affective Disorders and Schizophrenia for School-Age Children-Present and Lifetime Version) [34] was administered with both parents and the child as informant in order to establish the diagnosis of OCD and to assess past and current psychiatric comorbidity. Patients with psychiatric comorbidities other than OCD were not excluded. In addition, due to the naturalistic design of the study, we allowed the inclusion of patients receiving psychoactive medications. Exclusion criteria included intellectual disability, neurological disorders, and known inflammatory disease. The age of onset of OCD was defined as the age at which patients first displayed significant distress or impairment associated with obsessive-compulsive symptoms. OCD severity was measured at the time of admission using the Children’s Yale-Brown Obsessive-Compulsive Scale (CY-BOCS) [35], whose maximum score is 40 points.

Forty-seven healthy controls (age range 11–18 years) were recruited from schools in the same geographical region. Controls and their parents were interviewed with the Spanish version [33] of the K-SADS-PL to assess current and past psychopathology. Subjects with a personal history of psychiatric disorders, intellectual disability, and other neurological illness or known inflammatory disease were excluded.

All subjects were recruited between 2010 and 2014. All participants denied the use of alcohol or other recreational drugs during the semi-structured interview. All procedures were approved by the hospital’s ethics committee. Written informed consent was obtained from all parents and verbal informed consent was given by all subjects following an explanation of the procedures involved.

### Isolation of peripheral blood mononuclear cell (PBMC)

Blood samples of both OCD patients and healthy individuals were collected in BD Vacutainer tubes containing acid citrate dextrose (Becton Dickinson, Franklin Lakes, New Jersey, USA) for immune cell preparation. All samples were taken during the morning, before 12 p.m. Peripheral blood mononuclear cell (PBMC) suspensions were prepared by density gradient centrifugation over Ficoll-Plaque (GE Healthcare Bio-Science AB, Uppsala, Sweden) at 750×*g* for 20 min at 18 °C. After washing the interphase cells with phosphate-buffered saline (PBS), PBMCs were frozen in fetal bovine serum (FBS) (Life Technologies, Carlsbad, CA, USA) containing 10% dimethyl sulfoxide (Sigma-Aldrich, St. Louis, MO, USA) and stored in liquid nitrogen until subsequent trials in order to test patient and control immune cells in the same series of experiments and thus to avoid batch effects/interassay variation.

For subsequent analysis, cryopreserved PBMCs were rapidly thawed and washed with RPMI 1640 supplemented with 2 mM L-glutamine, 10% FBS, 100 units/mL penicillin, 100 μg/mL streptomycin (Life Technologies, Carlsbad, CA, USA), and 50 units/mL Benzonase® nuclease (Sigma-Aldrich, Saint Louis, MO, USA). Cells were counted using an Ac·T diff™ automated analyzer (Beckman Coulter, Miami, FL, USA).

### Flow cytometric analysis

To investigate whether the composition of peripheral monocytes is altered in early-onset OCD, a FACS-based analysis was performed. For this purpose, an aliquot of thawed PBMC containing at least 50,000 cells was washed with PBS and stained with fixable viability dye (eBioscience, San Diego, CA, USA) for 10 min at room temperature, followed by surface staining for 20 min with anti-HLA-DR-V500, anti-CD14-Pe-Cy7, and anti-CD16-FITC antibodies (BD Pharmingen, San Diego, CA, USA). After incubation, cells were analyzed by flow cytometry on a BD FACSCanto II (BD Bioscience, San Jose, CA, USA) using appropriate color compensation to correct for spectral overlap and autofluorescence. Data were analyzed with FlowJo version 10.1r5 software (Ashland, OR, USA). The gating strategy for identification of monocyte subsets is shown in Additional file 1: Figure S1. Firstly, the putative monocyte population was gated based on forward and side scatter profiles. Then, after selecting viable cells, monocytes were discriminated from the rest of leukocytes based on high expression of HLA-DR. Finally, the different patterns of expression of CD14 and CD16 allowed the identification of the three monocyte subsets: classical (CD14^high^CD16−), intermediate (CD14^high^CD16^low^), and non-classical (CD14^low^CD16^high^) monocytes. Isotype-matched control antibodies were used to determine the cutoff between negative and positive CD16. The combination of intermediate and non-classical monocytes in a single population was considered as CD16+ monocytes. Total monocytes were considered as the sum of classical, intermediate, and non-classical monocytes. Percentages of total monocytes, classical, intermediate, and non-classical monocyte subsets, and all CD16+ monocytes were assessed. Only samples with at least 200 total monocytes were included in the statistical analysis (91 early-onset OCD and 34 healthy controls).

### Purification, culture, and stimulation of peripheral monocytes

For the subsequent assessment of monocyte functionality, monocytes were purified from PBMC by negative selection using an indirect magnetic labeling system (MACS, Miltenyi Biotec, Auburn, CA, USA) following the manufacturer’s instructions. Briefly, non-monocytes, such as T cells, NK cells, B cells, and dendritic cells, were labeled using a cocktail of biotin-conjugated antibodies and anti-biotin microbeads. These magnetically labeled non-monocytes were depleted by being retained on a MACS® Column in the magnetic field of a MACS Separator while the unlabeled monocytes passed through the column. This kit allowed the simultaneous enrichment of classical, intermediate, and non-classical monocytes. Purity of isolated monocytes was checked by flow cytometry (routinely > 95). No monocytes were purified from one sample from an OCD patient and one from a healthy control, despite following the same protocol.

Purified monocytes (101 OCD patients and 46 controls) were seeded at a density of 1.5 × 10^5^ cells/well on 24-well plates and allowed to rest for 2 h in RPMI 1640 supplemented with 2 mM L-glutamine, 10% FBS, 100 units/mL penicillin, and 100 μg/mL streptomycin (Life Technologies, Carlsbad, CA, USA) in a humidified incubator with 5% CO_2_ at 37 °C prior to stimulation. To characterize monocyte functionality, monocytes were exposed to lipopolysaccharide (LPS), which is the major component of the outer membrane of Gram-negative bacteria, in order to stimulate the immune response, and dexamethasone, a glucocorticoid known to have immunosuppressive properties, to test monocyte sensitivity to immunoregulation. Hence, after resting for 2 h, monocytes were preincubated for 30 min with or without 100 nM dexamethasone followed by stimulation for 24 h with 1 ng/mL LPS (Sigma-Aldrich, Saint Louis, MO, USA). LPS was dissolved in culture medium whereas a stock solution of dexamethasone (1 mg/mL) was prepared in ethanol and further diluted in culture medium. Ethanol had no effect on monocyte cultures at the concentration used in the experiment (final ratio of 1/25000). Hence, sterile culture medium was used as negative control for all conditions. After 24 h of incubation, a supernatant was collected and cells were frozen. Supernatants were stored at − 20 °C until subsequent analysis.

### Cytokine quantification in cell culture supernatant

For the analysis of monocyte functionality, inflammatory cytokine levels in culture supernatants were measured in basal conditions and after LPS or dexamethasone plus LPS exposure of purified monocytes using multiplex bead-based sandwich immunoassay with Luminex xMAP® technology (Luminex, Austin, TX, USA). A commercially available 5-plex panel including proinflammatory cytokines and chemokines (IL-1β, IL-6, granulocyte-macrophage colony-stimulating factor (GM-CSF), TNF-α, and IL-8) (cat no. LHC0003, Invitrogen, Carlsbad, CA, USA) was used following the manufacturer’s instructions. Briefly, supernatants (25 μL) and standards were incubated with cytokine-specific capture antibodies coupled to fluorescent beads. Then, biotinylated detector antibodies were added, followed by streptavidin-phycoerythrin incubation. All standards and samples were analyzed in duplicate. Data were acquired using Luminex 200 system and analyzed with xPonent v3.1 software (Luminex, Austin, TX, USA). Cytokine levels were quantified using a five-parameter logistic regression curve derived from the reference cytokine concentration standards supplied by the manufacturer. The sensitivity of the assay allowed the detection of cytokine concentrations within the following ranges: IL-1β 0.33–6800 pg/mL, IL-6 0.25–5000 pg/mL, GM-CSF 0.77–15,400 pg/mL, TNF-α 0.36–7300 pg/mL, IL-8 0.47–9500 pg/mL. For experimental data above the range of the standard curve, the values were extrapolated, whereas samples below the lower limit of quantitation were assigned a value of one half of the minimum detection level for that cytokine as reported in previous studies [36, 37]. For the vast majority of the cytokines and assay conditions, at least 80% of the samples were within the limits of quantification. For some samples, it was not possible to determine cytokine concentration and so the following numbers of samples were included in the statistical analysis for each cytokine: IL-1β, 100 OCD and 43 controls; IL-6, 79 OCD and 39 controls; GM-CSF, 100 OCD and 44 controls; TNF-α, 98 OCD and 44 controls; and IL-8, 79 OCD and 40 controls.

In order to assess inflammatory activation of peripheral monocytes upon an immune challenge, percentages of cytokine production with respect to untreated sample were calculated for each cytokine level after treatment with LPS alone or LPS plus dexamethasone. Besides, to estimate monocyte sensitivity to dexamethasone, differences in cytokine concentrations between LPS-treated and LPS + dexamethasone-treated samples were computed and expressed as percentages with respect to LPS stimulation.

### Statistical analysis

Data were analyzed using IBM SPSS statistics 20 (IBM Corp., Chicago, IL, USA). Normality was assessed using Kolmogorov-Smirnov and Shapiro-Wilk tests. The different immune parameters, including all percentages of monocyte subsets and the five cytokine levels in basal conditions and after LPS or LPS + dexamethasone treatment, were found to follow a non-normal distribution. Therefore, all variables were natural-log-transformed for subsequent analysis. In order to test differences in sociodemographic variables between patients and controls, Student’s *t* test for continuous variables and chi-square test for categorical variables were used. Pearson’s test was also used to assess correlations. Univariate analyses were performed adjusting for gender and age to assess the difference in immune parameters between patients and controls. When a significant correlation was identified between cytokine levels and monocyte subsets, the corresponding monocyte subpopulation was introduced as a covariate in the univariate analysis. When the immune parameters were found to be associated with OCD, we also assessed their putative relationship with clinical variables such as disease severity, duration of disease, or psychopharmacological status. When appropriate, further pairwise comparison analyses were performed using Bonferroni’s post hoc test.

All data are reported as means ± standard error of the mean (SEM) of the original data, prior to log transformation. All tests were two-tailed and significance level was set at *p* < 0.05.

## Results

### Clinical and sociodemographic characteristics of the sample

Demographic and clinical data of patients with OCD and the control group are shown in Table 1. The mean age was 14.78 years for OCD individuals and 16.29 years for healthy controls (*t* = − 3.608, *p* = 0.0004). There were 52 boys (51%) and 50 girls (49%) in the case group and 14 boys (29.8%) and 33 (70.2%) girls among the controls (*χ* ^2^ = 5.86, *p* = 0.016). The mean symptom severity score in OCD individuals, as assessed by the CY-BOCS scale was 25.97 ± 0.64; this high value reflected the pathological condition of patients. Some patients were also diagnosed with internalizing disorders (anxiety or depression disorders) (*N* = 46, 45.1%) or with neurodevelopmental disorders (attention deficit hyperactivity disorder (ADHD) or tics) (*N* = 22, 21.6%). However, the presence of these comorbidities in some OCD patients does not seem to have an effect on the frequency of monocyte subsets and cytokine levels, as no differences were found between the groups (Additional file 2: Table S1).

**Table 1:** Demographic and clinical data of the study population

	OCD patients (*N* = 102)	Controls (*N* = 47)	Statistic	*p* value
Male gender, *N* (%)	52 (51.0)	14 (29.8)	*χ* ^2^ = 5.86	0.016^a^
Age (mean ± SEM)	14.78 ± 0.26	16.29 ± 0.26	*t* = − 3.608	0.0004^b^
Age of onset (mean ± SEM)	12.95 ± 0.28	–	–	–
Duration of illness, months (mean ± SEM)	24.62 ± 2.44	–	–	–
CY-BOCS score (mean ± SEM)	25.97 ± 0.64	–	–	–
Comorbidities, *N* (%)				
Anxiety or mood disorders	46 (45.1)	–	–	–
ADHD or tic disorder	22 (21.6)	–	–	–
Treatment, *N* (%)				
Medicated	82 (80.4)	–	–	–
Antidepressants	59 (72)	–	–	–
Antipsychotics	1 (1.2)	–	–	–
Antidepressants + antipsychotics	22 (26.8)	–	–	–
Non-medicated	20 (19.6)	–	–	–

*OCD* obsessive compulsive disorder, *ADHD* attention deficit hyperactivity disorder, *CY-BOCS* Children’s Yale-Brown Obsessive-Compulsive Scale, *SEM* standard error of the mean

^a^Chi-square test

^b^Student’s *t* test

Eighty-two patients (80.4%) received pharmacological treatment with an antidepressant alone (*N* = 59, 72%), an antidepressant combined with antipsychotic drugs (*N* = 22, 26.8%), or an antipsychotic alone (*N* = 1, 1.2%). The most prescribed antidepressant was fluoxetine (in 45.7% of patients receiving antidepressants), whereas the most used antipsychotic was risperidone (in 43.5% of patients taking antipsychotics). Ten patients were comedicated with benzodiazepines (12.2%), four (4.9%) with long-acting methylphenidate, and one (1.2%) with lithium carbonate.

Although a different number of samples was included in the analysis of monocyte subsets and the five proinflammatory cytokines, no differences were identified between these populations regarding any of the clinical and sociodemographic characteristics (data not shown).

### Enrichment of CD16+ monocytes in peripheral blood of OCD patients

**Fig. 1:**
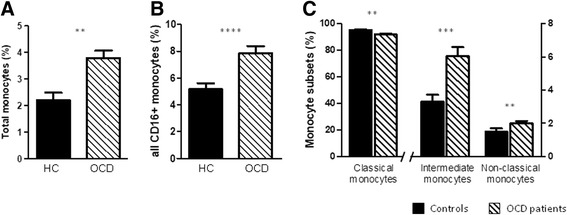
Distribution of monocyte subsets in early-onset OCD patients (*N* = 91) and healthy controls (HC, *N* = 34). **a** Percentage of total monocytes in OCD patients and healthy controls. **b** Percentage of all CD16+ monocytes, including CD14^high^CD16^low^ and CD14^low^CD16^high^ monocytes, in OCD patients and healthy controls. **c** Percentage of classical (CD14^high^CD16−), intermediate (CD14^high^CD16^low^), and non-classical (CD14^low^CD16^high^) monocytes in OCD patients and healthy controls. Results are expressed as means ± SEM of the original data, prior to log transformation. Statistical analysis was performed using univariate general linear model adjusted for age and gender with natural-log-transformed data. **p < 0.01, ***p < 0.001, ****p < 0.0001

OCD patients presented higher percentages of total monocytes than healthy controls (*F* = 7.199, *p* = 0.008) (Fig. 1a). Regarding different monocyte subsets, CD16+ monocytes were enriched in OCD patients compared to healthy controls (*F* = 18.188, *p* = 0.00004) (Fig. 1b). This effect was largely driven by a twofold increase in intermediate monocytes in OCD patients (*F* = 15.013, *p* = 0.0002) (Fig. 1c), although non-classical monocytes were also slightly higher in OCD individuals (*F* = 7.107, *p* = 0.009). Concurrently, classical monocytes were slightly less frequent in patients than in controls (*F* = 10.188, *p* = 0.002) (Fig. 1c).

### Higher production of inflammatory cytokines in OCD patients

There was a high correlation between the levels of the five different cytokines measured in the study (Additional file 3: Table S2). In addition, cytokine levels were mainly significantly correlated with total monocytes but not with the different monocyte subsets (Additional file 4: Table S3).

**Fig. 2:**
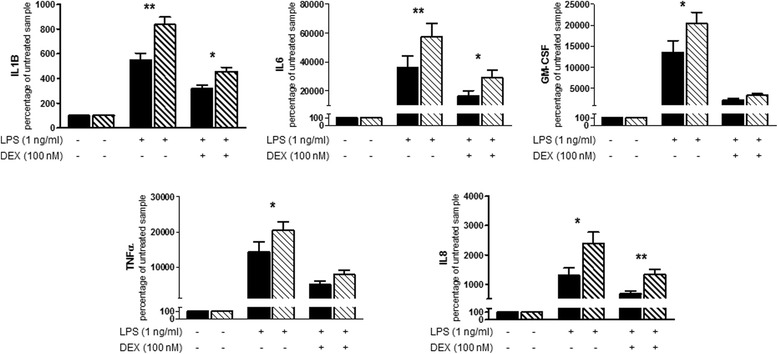
Cytokine secretion by purified monocytes of early-onset OCD and healthy controls. Monocytes were cultured for 24 h with vehicle, lipopolysaccharide (LPS) 1 ng/mL or pretreatment with dexamethasone (DEX) 100 nM for 30 min followed by addition of LPS 1 ng/mL. Cytokine secretion was assessed in culture supernatants using Luminex xMAP Technology. Due to limitations of the immunoassay sensitivity, the sample sizes may vary for each cytokine: IL-1β, 100 OCD and 43 controls; IL-6, 79 OCD and 39 controls; GM-CSF, 100 OCD and 44 controls; TNF-α, 98 OCD and 44 controls; and IL-8, 79 OCD and 40 controls. Results are expressed as means ± SEM of the original data, prior to log transformation. Statistical analysis was performed using univariate general linear model adjusted for age and gender with natural-log-transformed data.*p < 0.05, **p < 0.01

No differences were identified in cytokine levels in basal conditions between patients and controls (Additional file 5: Table S4). Effects of monocyte stimulation with LPS or LPS + dexamethasone are shown in Fig. 2. After LPS treatment, OCD samples showed a significantly higher production of inflammatory cytokines (IL-1β, *F* = 8.216 *p* = 0.005; IL-6, *F* = 8.570 *p* = 0.004; GM-CSF, *F* = 4.433 *p* = 0.049; TNF-α, *F* = 4.241 *p* = 0.041; IL-8, *F* = 6.330 *p* = 0.013) than healthy controls. As expected, cells pretreated with dexamethasone before LPS stimulation released lower quantities of inflammatory cytokines than those treated with LPS alone in both OCD patients and controls. However, cytokine levels after LPS + dexamethasone treatment were still higher than in basal conditions. Due to cytokine production after LPS stimulation, monocytes from OCD patients released higher quantities of the inflammatory cytokines than healthy controls after LPS + dexamethasone treatment. These differences were only significant for some of the mediators: IL-1β (*F* = 5.528; *p* = 0.020), IL-6 (*F* = 6.284; *p* = 0.014), GM-CSF (*F* = 2.205; *p* = 0.140), TNF-α (*F* = 1.576; *p* = 0.211), and IL-8 (*F* = 10.326; *p* = 0.002). Nevertheless, monocyte sensitivity to dexamethasone expressed as a percentage of reduction in cytokine levels in LPS + dexamethasone-treated monocytes with respect to LPS-stimulated cells did not differ between patients and controls (Additional file 5: Table S4).

### Clinical characteristics of OCD patients and immune parameters

**Fig. 3:**
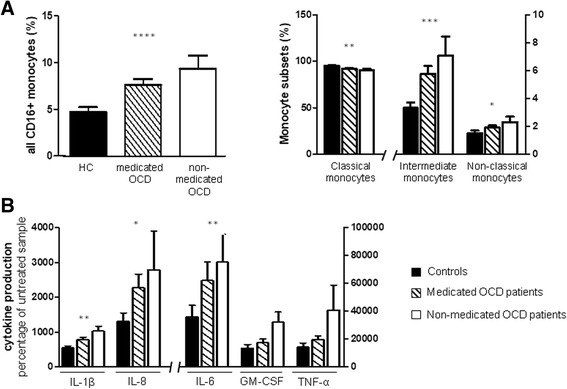
Influence of medication on immune parameters in OCD. **a** Distribution of monocyte subsets (percentages of all CD16+, classical, intermediate, and non-classical monocytes) in peripheral blood of medicated (*N* = 71) and non-medicated patients (*N* = 20) with early-onset OCD and healthy controls (HC, *N* = 34). **b** Cytokine secretion by purified monocytes of medicated and non-medicated patients with early-onset OCD and healthy controls after 24 h of stimulation with LPS 1 ng/mL (IL-1β 43 controls, 80 medicated OCD, 20 non-medicated OCD; IL-8 40 controls, 62 medicated OCD, 17 non-medicated OCD; IL-6 39 controls, 66 medicated-OCD, 13 non-medicated OCD; GM-CSF 44 controls, 80 medicated OCD, 20 non-medicated OCD; TNF-α 44 controls, 79 medicated OCD, 19 non-medicated OCD). Results are expressed as means ± SEM of the original data, prior to log transformation. Statistical analysis was performed using univariate general linear model adjusted for age and gender with natural-log-transformed data. *p < 0.05, **p < 0.01, ***p < 0.001, ****p < 0.0001

We also addressed the possible effects of pharmacological treatment on monocyte distribution and cytokine production in cultured monocytes. We found a progressive increase in CD16+ and intermediate and non-classical subsets from healthy subjects to OCD individuals receiving pharmacological treatment and finally to non-treated patients (CD16+ monocytes *F* = 10.372, *p* = 0.00007; intermediate monocytes *F* = 8.647, *p* = 0.0003; non-classical monocytes *F* = 3.561, *p* = 0.032) (Fig. 3a). In contrast, the direction of this association was reversed for classical monocytes, since the percentage of this subset was higher in healthy individuals than in treated patients and finally in unmedicated patients (*F* = 6.620, *p* = 0.002) (Fig. 3a). The same pattern was identified for cytokine levels after LPS stimulation: there was a progressive increase in cytokine concentrations from healthy subjects to OCD patients receiving pharmacological treatment and finally to patients without medication. This association was significant for most cytokines (IL-1β *F* = 5.464, *p* = 0.005; IL-6 *F* = 5.076, *p* = 0.008; GM-CSF *F* = 1.917, *p* = 0.151; TNF-α *F* = 2.102, *p* = 0.126; and IL8 *F* = 4.526, *p* = 0.013, Fig. 3b). Post hoc comparisons revealed that healthy controls had lower levels of monocyte subsets and proinflammatory cytokines compared to both untreated and treated OCD patients (*p* < 0.05). However, no significant differences were found between OCD individuals receiving psychoactive medications and untreated patients.

In addition, we evaluated the putative relationship of monocyte subset frequency and cytokine production with severity of obsessive-compulsive symptoms as well as with duration of disease. None of the immune parameters were significantly correlated with these clinical variables (Additional file 6: Table S5).

## Discussion

To our knowledge, this is the first study to evaluate the distribution of peripheral monocyte subsets and to analyze markers of monocyte activation and functionality in early-onset OCD. Our results revealed a proinflammatory predisposition of monocytes from OCD patients based on the imbalance of monocyte subpopulations and their over-reactivity to immune stimulation with LPS. Moreover, OCD patients receiving psychoactive medications were found to have an intermediate inflammatory profile compared to untreated patients and controls.

OCD patients showed an increase in the percentage of total monocytes as well as in the CD16+ subset, especially in intermediate monocytes but also in non-classical monocytes, compared with healthy controls. Although there is no previous evidence of an association between altered monocyte frequencies and OCD, some authors have identified high levels of monocytes in related disorders like Tourette’s syndrome (TS) [38] or autism spectrum disorder [39, 40]. However, no differences in the monocyte subset distribution have been found [41].

Together with these alterations in monocyte subsets, we found that isolated peripheral blood monocytes from children with OCD behaved abnormally upon stimulation with LPS, displaying excessive IL-1β, IL-6, GM-CSF, TNF-α, and IL-8 production compared to monocytes from healthy controls. Interestingly, we did not detect any abnormal activation of monocytes from OCD patients in basal conditions, but exposure to an immune challenge was required for the over-reactivity of these cells. Whether this over-reactivity occurs upon other types of immune stimulus such as psychological stress remains to be elucidated. These findings are at odds with results from previous studies with similar methodologies, which have reported either no differences or lower production of several cytokines after LPS stimulation in OCD individuals [42–45]. However, we should stress that in these studies, the assessments involved different types of samples including other immune cells and all of them were conducted in adult patients, in whom the pathological mechanism may be different [4, 5]. In addition, cytokine production in children has been reported to be different from that in adults [46, 47]. Apart from the studies mentioned above, most authors have investigated cytokine levels in serum or cerebrospinal fluid in OCD [16–18, 47, 48] but the results were inconsistent due to methodological and sample differences such as age, age at onset, disease duration, and pharmacological treatment. Interestingly, in our population, monocytes from pediatric OCD patients were able to respond to glucocorticoid exposure in the same way as those from healthy individuals. However, due to the over-reactivity of monocytes from OCD patients after LPS stimulation, cytokine levels remained higher in patients than in controls when treated with dexamethasone. The significant correlation identified between cytokine levels and the percentage of total monocytes indicates that those individuals with higher production of inflammatory cytokines, both in basal conditions and in response to stimulus, were also those with the higher levels of monocytes and thus with a greater global inflammatory state. However, as all the samples were cultured using the same amount of monocytes, we can rule out that the amount of cytokines released was due to the number of monocytes. In addition, as cytokine levels were mainly not correlated with the different monocyte subsets, the higher production of cytokines must be due to a higher activation of monocytes rather than to monocyte subset distribution.

This study also found that OCD patients receiving pharmacological treatment seem to present an intermediate inflammatory profile (including monocyte subset distribution and cytokine production upon stimulation with LPS) that is lower than non-medicated OCD individuals and higher than healthy controls. These results are in accordance with previous evidence suggesting that treatment with antidepressant medications may have anti-inflammatory properties [49, 50]. However, post hoc analysis was not able to detect significant differences between treated and untreated patients, maybe due to the small size of the non-medicated group. If the trend identified here is confirmed in subsequent studies, the combined treatment with antidepressants and anti-inflammatory drugs like glucocorticoids may be a promising therapy for improving obsessive-compulsive symptoms, as observed in patients with major depressive disorder or schizophrenia [51, 52]. In addition, other immune-modulating medications, such as anticytokine agents, may be useful as adjunct therapy in OCD individuals, as observed in a patient with tumor necrosis factor receptor-associated autoinflammatory syndrome (TRAPS), in which psychiatric symptoms, including tics and OCD, showed a remarkable improvement after treatment with the IL-1 blocking agent anakinra [53].

Monocytes, which were found to be increased in OCD patients in the present study, are the main cells implicated in the first immune response upon infection. Among the different monocyte subsets, CD16+ and specifically the intermediate monocytes, which were increased in our OCD population, are considered the most inflammatory subpopulation due to their expression of inflammatory cytokines and other activation markers and due to their expansion in infectious and inflammatory diseases [29–31]. Circulating monocytes can be recruited to the central nervous system (CNS) upon exposure to psychosocial stress [25] or when chronic or intense injury occurs in the brain, where they contribute to the inflammatory response with their phagocytic activity and the release of immune mediators such as cytokines [28, 54]. Additionally, peripherally produced cytokines secreted by monocytes, such as the IL-1β, IL-6, GM-CSF, TNF-α, and IL-8 evaluated in this study, not only act peripherally but also enter the CNS where they increase neuroinflammatory responses and affect neurotransmitter availability [55, 56], brain function, and neurodevelopment [57]. These effects occur mainly in the basal ganglia and dorsal anterior cingulate cortex [58], which have been implicated in the pathophysiology of OCD [59]. Hence, monocyte activation upon immune challenges and other potential stressors in genetically susceptible individuals may be involved in the onset, progression, and exacerbation of obsessive-compulsive symptoms, as proposed for other psychiatric diseases such as schizophrenia [23, 60], depression [58] or anxiety [21]. This peripheral activation of monocytes may also suggest microglial activation, since they are the innate immune cells of the CNS. Although microglia originate from myeloid precursors deriving from the yolk sac during embryonic development, with minimal contribution of bone marrow-derived progenitors in adulthood [54, 61], monocytes and microglial cells may exhibit similar responses to systemic stimuli [54, 62]. Activated microglia with a proinflammatory phenotype are unable to remove debris and promote regeneration of the inflamed tissue leading to a failure in immune resolution and neuroprotection [28]. In addition, microglial abnormalities may lead to alterations of synaptic pruning as well as higher release of microglial-derived glutamate, which in turn might have neurotoxic effects on dendrites and synapses [63]. Indeed, it has been suggested that microglial dysregulation may have a role in the pathophysiology of OCD and related diseases like TS or autism. *Postmortem* studies evaluating gene expression in basal ganglia from TS subjects identified an increase in the expression of monocyte chemotactic factor-1 (MCP-1) [64] and an upregulation of several microglia-related genes [65], pointing to microglia proliferation and activation. In addition, animal models such as *Hoxb8* knockout mice or *L-histidine decarboxylase (Hdc*) knockout mice, exhibiting repetitive behaviors, mostly grooming, have also suggested the participation of microglia in the development of OCD and TS [63, 66, 67].

Taken together, our results indicate an enhanced proinflammatory state in monocytes in early-onset OCD characterized by alterations in monocyte subset distribution as well as higher production of inflammatory cytokines after monocyte stimulation. It should be borne in mind that our sample comprised OCD patients with a variety of comorbidities and that most patients were receiving pharmacological treatment. However, the higher activation state of monocytes from OCD patients identified here was not due to the presence of these conditions. We should also stress the homogeneity of our OCD sample in terms of age, age at onset, or duration of disease, as all participants were children and adolescents; this avoids the possible influence of these confounding factors and allows the study of the specific etiopathogenic mechanism underlying childhood-onset OCD, which may differ from the pathophysiology of the adult disorder [5]. In addition, a larger size of the control sample, at least similar to that of the OCD group, would be preferred. However, due to the frequent difficulties found in the recruitment of healthy children and adolescents, we were not able to include a higher number of controls. Moreover, despite the imbalance in gender between OCD patients and healthy controls and the age difference between the groups, the results of the univariate analysis adjusted for these two factors revealed that the association between OCD and immune parameters that was identified here was independent of their potential confounding effect.

As we used a primary model of isolated monocytes, the enhanced proinflammatory state in OCD patients shown in this study was not influenced by the immune response mediated by other immune cells. This means that we can specifically determine the potential contribution of monocyte lineage cells to the inflammatory response in early-onset OCD. Nevertheless, although the over-activation of monocytes found in the present study was demonstrated by a number of parameters, only peripheral activation markers were measured. Hence, even though peripheral monocytes and circulating cytokines are able to enter the brain [55] and although these peripheral changes may also reflect microglial activation [23], analysis of more inflammatory markers, including central immune changes, is needed in order to understand the complex inflammatory profile underlying OCD. In addition to these limitations, it should also be borne in mind that no adjustments for multiple comparisons were applied due to the exploratory nature of the study and the high correlation of the monocyte-derived products. Hence, the results should be interpreted with caution.

## Conclusions

In conclusion, the results of the present study point to a proinflammatory state of monocytes from children and adolescents with OCD. The enhanced activation of the innate immune system may be triggered by exposure to immunomodulatory stimuli like stress or infections leading to an increased production of cytokines that mediate the inflammatory process. Further studies are necessary to elucidate the role of innate immunity in the complex pathophysiology of OCD and to understand the interplay between neural and immune cells throughout the different stages of development.

## Additional files

Additional file 1: Figure S1. Gating strategy for identification of monocyte subpopulations. (DOCX 324 kb)

Additional file 2: Table S1. Analysis of the percentages of total monocytes, monocyte subpopulations, and cytokine levels after LPS stimulation of purified monocytes in early-onset OCD diagnosed with different comorbidities. (DOCX 22 kb)

Additional file 3: Table S2. Correlations between the five proinflammatory cytokines measured in the study in basal conditions and after LPS or LPS-dexamethasone stimulation. (DOCX 21 kb)

Additional file 4: Table S3. Correlations between the monocyte subsets and the levels of the five proinflammatory cytokines measured in the study in basal conditions and after LPS or LPS-dexamethasone stimulation. (DOCX 23 kb)

Additional file 5: Table S4. Cytokine secretion by purified monocytes of early-onset OCD and healthy controls. (DOCX 24 kb)

Additional file 6: Table S5. Correlations of the duration of disease (expressed in months) and symptom severity (assessed by CY-BOCS score), with the percentage of total monocytes, monocyte subpopulations, and cytokine levels after LPS stimulation of monocytes in early-onset OCD. (DOCX 13 kb)

## Abbreviations

ADHD: Attention deficit hyperactivity disorder
CNS: Central nervous system
CY-BOCS: Children’s Yale-Brown obsessive-compulsive scale
DSM-IV: Diagnostic and Statistical Manual of Mental Disorders
FBS: Fetal bovine serum
GM-CSF: Granulocyte-macrophage colony-stimulating factor
Il-1: Interleukin 1
IL-1β: Interleukin 1 beta
IL-6: Interleukin 6
IL-8: Interleukin 8
K-SADS-PL: Schedule for affective disorders and schizophrenia for school-age children-present and lifetime version
LPS: Lipopolysaccharide
OCD: Obsessive-compulsive disorder
PANDAS: Pediatric Autoimmune Neuropsychiatric Disorders Associated with Streptococcal Infections
PBMC: Peripheral blood mononuclear cell
PBS: Phosphate buffer saline
SC: Sydenham’s chorea
TNF-α: Tumor necrosis factor alpha
TRAPS: Tumor necrosis factor receptor-associated autoinflammatory syndrome
TS: Tourette’s syndrome
