# Supplementary material for: Inflammatory dysregulation of monocytes in pediatric patients with obsessive-compulsive disorder
Source: J Neuroinflammation. 2017 Dec 28;14:261. doi: 10.1186/s12974-017-1042-z (PMC5746006; doi:10.1186/s12974-017-1042-z)
Supplement: Supplementary file 1 — Gating strategy for identification of monocyte subpopulations. (DOCX 324 kb) [file 12974_2017_1042_MOESM1_ESM.docx]

**Figure S1**


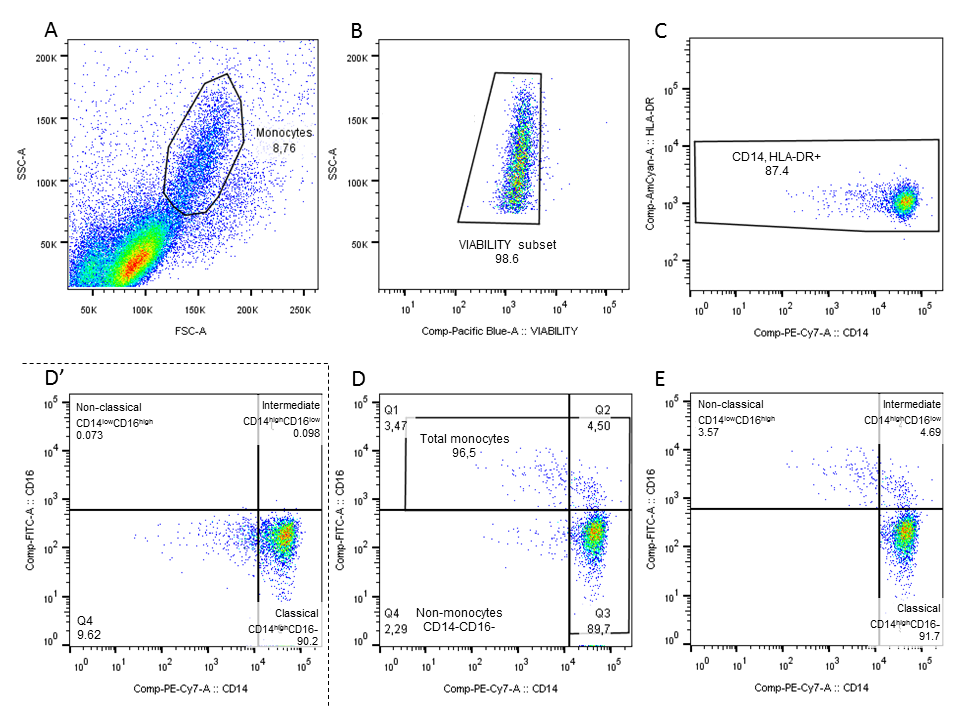


Figure S1: Gating strategy for identification of monocyte subpopulations.

After gating the putative monocyte population on the basis of forward and side scatter profiles (A), viable cells were selected (B). Then, cells were displayed on a HLA-DR and CD14 plot and a gate was set on HLA-DR positive cells (C). Monocyte subpopulations were identified from this gate based on their differential expression of CD14 and CD16 in a dot plot. Non-monocyte cells (CD14-, CD16-) were excluded (D). A vertical line was placed at the left-hand end of CD14 staining of the classical monocytes. An isotype control was used to determine the cutoff between negative and positive CD16 (D’). CD14^high^CD16- cells were considered as classical monocytes, CD14^high^CD16^low^ were taken as intermediate monocytes and CD14^low^CD16^high^ were considered as non-classical monocytes (E). The combination of intermediate and non-classical monocytes in a single population was considered as CD16+ monocytes. Frequency of each monocyte subset is expressed as percentage of total monocytes (classical + intermediate + non-classical subsets).
