# Supplementary material for: Inflammatory dysregulation of monocytes in pediatric patients with obsessive-compulsive disorder
Source: J Neuroinflammation. 2017 Dec 28;14:261. doi: 10.1186/s12974-017-1042-z (PMC5746006; doi:10.1186/s12974-017-1042-z)
Supplement: Supplementary file 2 — Analysis of the percentages of total monocytes, monocyte subpopulations, and cytokine levels after LPS stimulation of purified monocytes in early-onset OCD diagnosed with different comorbidities. (DOCX 22 kb) [file 12974_2017_1042_MOESM2_ESM.docx]

**Table S1.** Analysis of the percentages of total monocytes, monocyte subpopulations and cytokine levels after LPS stimulation of purified monocytes in early-onset OCD diagnosed with different comorbidities.

|  | OCD patients  without comorbidities | |  | OCD patients with anxiety or mood disorders | |  | OCD patients with ADHD and/or tics | | Statistic*F* | p-value |
| --- | --- | --- | --- | --- | --- | --- | --- | --- | --- | --- |
|  | N | Mean ±SEM |  | N | Mean ±SEM |  | N | Mean ±SEM |  |  |
| **Total monocytes and monocyte subsets** | | |  |  |  |  |  |  |  |  |
| Total monocytes (%) | 33 | 4.01 ± 0.35 |  | 40 | 4.51 ± 0.49 |  | 18 | 3.76 ± 0.76 | 0.20 | 0.818 |
| CD16+ monocytes^a^ | 33 | 6.33 ± 1.08 |  | 40 | 7.64 ± 0.77 |  | 18 | 8.02 ± 1.38 | 0.24 | 0.787 |
| Classical monocytes^a^ | 33 | 91.44 ± 1.08 |  | 40 | 92.24 ± 0.77 |  | 18 | 91.91 ± 1.37 | 0.21 | 0.814 |
| Intermediate monocytes^a^ | 33 | 6.48 ± 0.97 |  | 40 | 5.50 ± 0.72 |  | 18 | 6.47 ± 1.37 | 0.48 | 0.621 |
| Non-classical monocytes^a^ | 33 | 2.04 ± 0.23 |  | 40 | 2.14 ± 0.26 |  | 18 | 1.55 ± 0.18 | 0.39 | 0.676 |
| **Cytokine secretion after LPS stimulation (% of basal conditions)** | | | | | |  |  |  |  |  |
| IL-1β | 34 | 815.05 ± 77.36 |  | 44 | 877.57 ± 108.02 |  | 22 | 789.02 ± 136.18 | 0.40 | 0.635 |
| IL-6 | 26 | 59 735.15 ± 15 067.94 |  | 31 | 67 398.48 ± 18 659.10 |  | 22 | 40 470.45 ± 13 041.83 | 0.50 | 0.254 |
| GM-CSF | 34 | 23 709.67 ± 5 018.21 |  | 44 | 21 401.99 ± 4 041.89 |  | 22 | 13 759.87 ± 3 539.09 | 1.73 | **0.024^#^** |
| TNF-α | 34 | 20 488.82 ± 3 705.17 |  | 42 | 22 797.90 ± 4 255.25 |  | 22 | 15 937.19 ± 3 925.21 | 0.27 | 0.312 |
| IL-8 | 28 | 2 315.44 ± 516.20 |  | 34 | 1 672.99 ± 267.05 |  | 17 | 3 948.24 ± 1 458.22 | 0.63 | 0.902 |

^a^ Expressed as percentage of total monocytes. Statistical analysis was performed using univariate general linear model with natural-log-transformed data. Results are expressed as means ± SEM of the original data, prior to log-transformation.

Significant results are shown in bold. ^#^ Post-hoc comparisons revealed significant differences between OCD patients with ADHD and/or tics and those without comorbidities (p=0.020) as well as between OCD comorbid with ADHD and/or tics and OCD comorbid with anxiety and/or mood disorders (p=0.033)
