# Supplementary material for: Inflammatory dysregulation of monocytes in pediatric patients with obsessive-compulsive disorder
Source: J Neuroinflammation. 2017 Dec 28;14:261. doi: 10.1186/s12974-017-1042-z (PMC5746006; doi:10.1186/s12974-017-1042-z)
Supplement: Supplementary file 3 — Correlations between the five proinflammatory cytokines measured in the study in basal conditions and after LPS or LPS-dexamethasone stimulation. (DOCX 21 kb) [file 12974_2017_1042_MOESM3_ESM.docx]

**Table S2.** Correlations between the five proinflammatory cytokines measured in the study in basal conditions and after LPS or LPS-dexamethasone stimulation.

|  |  | IL-1β | IL-6 | GM-CSF | TNF-α | IL-8 |  |
| --- | --- | --- | --- | --- | --- | --- | --- |
|  | N total | 143 | 117 | 143 | 141 | 118 |  |
|  | IL-1β |  | R=0.803; **p=1.2 x 10^-27^** | R=0.668; **p=8.1 x 10^-20^** | R=0.832; **p=2.1 x 10^-^**^37^ | R=0.946; **p=1.3 x 10^-^**^58^ | **Basal cytokine production (pg/ml)** |
| **Cytokine production after LPS stimulation**  **(% of basal conditions)** | IL-6 | R=0.672; **p=1.1 x 10^-16^** |  | R=0.671; **p=9.5 x 10^-17^** | R=0.891; **p=1.7 x 10^-41^** | R=0.852; **p=3.7 x 10^-30^** |  |
|  | GM-CSF | R=0.559; **p=4.2 x 10^-13^** | R=0.615; **p=1.3 x 10^-13^** |  | R=739; **p=8.4 x 10^-26^** | R=0.630; **p=1.7 x 10^-14^** |  |
|  | TNF-α | R=0.638; **p=1.9 x 10^-17^** | R=0.849; **p=6.4 x 10^-34^** | R=0.678; **p=1.8 x 10^-20^** |  | R=0.858; **p=4.8 x 10^-35^** |  |
|  | IL-8 | R=0.550; **p=1.2 x 10^-10^** | R=0.610; **p=8.3 x 10^-12^** | R=0.517; **p=1.7 x 10^-9^** | R=0.549; **p=1.5 x 10^-10^** |  |  |
|  |  |  |  |  |  |  |  |
|  | IL-1β |  | R=0.591; **p=2.2 x 10^-12^** | R=0.561; **p=3.2 x 10^-13^** | R=0.674; **p=5.0 x 10^-20^** | R=0.762; **p=1.2 x 10^-23^** | **Cytokine production after LPS + dexamethasone treatment**  **(% of basal conditions)** |
| **Sensitivity to dexamethasone^a^** | IL-6 | R=0.565; **p=8.8 x 10^-11^** |  | R=0.550; **p=1.1 x 10^-10^** | R=0.805; **p=4.1 x 10^-28^** | R=0.654; **p=6.5 x 10^-14^** |  |
|  | GM-CSF | R=0.331; **p=7.2 x 10^-5^** | R=0.398; **p=1.2 x 10^-5^** |  | R=0.650; **p=2.0 x 10^-18^** | R=0.635; **p=9.3 x 10^-15^** |  |
|  | TNF-α | R=0.417; **p=4.4 x 10^-7^** | R=0.556; **p=1.1 x 10^-10^** | R=0.250; **p=0.003** |  | R=0.632; **p=2.2 x 10-^14^** |  |
|  | IL-8 | R=-0.075; p=0.461 | R=0.455; **p=6.0 x 10^-6^** | R=0.187; p=0.061 | R=0.142; p=0.160 |  |  |
|  |  |  |  |  |  |  |  |

^a^ Measured as percentage of reduction in cytokine levels in LPS+dexamethasone-treated monocytes with respect to LPS-stimulated.

Correlations were performed using the Pearson's correlation test.

Significant results are shown in bold.
