# Supplementary material for: Inflammatory dysregulation of monocytes in pediatric patients with obsessive-compulsive disorder
Source: J Neuroinflammation. 2017 Dec 28;14:261. doi: 10.1186/s12974-017-1042-z (PMC5746006; doi:10.1186/s12974-017-1042-z)
Supplement: Supplementary file 4 — Correlations between the monocyte subsets and the levels of the five proinflammatory cytokines measured in the study in basal conditions and after LPS or LPS-dexamethasone stimulation. (DOCX 23 kb) [file 12974_2017_1042_MOESM4_ESM.docx]

**Table S3.** Correlations between the monocyte subsets and the levels of the five proinflammatory cytokines measured in the study in basal conditions and after LPS or LPS-dexamethasone stimulation.

|  |  | Total monocytes (%) | CD16+ monocytes^a^ | Classical monocytes^a^ | Intermediate monocytes^a^ | Non-classical monocytes^a^ |
| --- | --- | --- | --- | --- | --- | --- |
| **Basal cytokine production (pg/ml)** | IL-1β | R=0.323;  **p=0.0003** | R=0.095;  p=0.302 | R=-0.023;  p=0.800 | R=0.077;  p=0.401 | R=0.087;  p=0.344 |
|  | IL-6 | R=0.276;  **p=0.006** | R=0.126;  p=0.220 | R=0.039;  p=0.707 | R=0.077;  p=0.458 | R=0.167;  p=0.103 |
|  | GM-CSF | R=136;  p=0.136 | R=0.196;  **p=0.031** | R=-0.222;  **p=0.014** | R=0.222;  **p=0.014** | R=0.005;  p=0.952 |
|  | TNF-α | R=0.267;  **p=0.003** | R=0.099;  p=0.283 | R=-0.026;  p=0.781 | R=0.058;  p=0.526 | R=0.105;  p=0.254 |
|  | IL-8 | R=0.190;  p=0.058 | R=-0.045;  p=0.658 | R=0.132;  p=0.189 | R=-0.111;  p=0.270 | R=0.098;  p=0.332 |
|  |  |  |  |  |  |  |
| **Cytokine production after LPS stimulation**  **(% of basal conditions)** | IL-1β | R=0.173;  p=0.057 | R=0.102;  p=0.265 | R=-0.017;  p=0.852 | R=0.023;  p=0.799 | R=0.176;  p=0.053 |
|  | IL-6 | R=0.133;  p=0.198 | R=0.078;  p=0.448 | R=-0.052;  p=0.615 | R=0.026;  p=0.800 | R=0.144;  p=0.160 |
|  | GM-CSF | R=0.446;  **p=2.6 x 10^-7^** | R=0.061;  p=0.507 | R=0.116;  p=0.203 | R=0.012;  p=0.895 | R=0.150;  p=0.100 |
|  | TNF-α | R=0.281;  **p=0.002** | R=0.047;  p=0.607 | R=0.054;  p=0.560 | R=-0.007;  p=0.944 | R=0.159;  p=0.083 |
|  | IL-8 | R=0.310;  **p=0.002** | R=0.178;  p=0.076 | R=-0.178;  p=0.076 | R=0.207;  **p=0.039** | R=0.061;  p=0.549 |
|  |  |  |  |  |  |  |
| **Cytokine production after LPS + dexamethasone treatment**  **(% of basal conditions)** | IL-1β | R=0.163;  p=0.073 | R=0.055;  p=0.548 | R=0.033;  p=0.723 | R=0.000;  p=0.999 | R=0.149;  p=0.104 |
|  | IL-6 | R=0.127;  p=0.219 | R=0.041;  p=0.695 | R=-0.015;  p=0.884 | R=-0.004;  p=0.969 | R=0.118;  p=0.254 |
|  | GM-CSF | R=0.349;  **p=8.5 x 10^-5^** | R=-0.13;  p=0.888 | R=0.193;  **p=0.034** | R=-0.064;  p=0.485 | R=0.131;  p=0.151 |
|  | TNF-α | R=0.190;  **p=0.038** | R=-0.040;  p=0.666 | R=0.161;  p=0.080 | R=-0.88;  p=0.337 | R=0.126;  p=0.169 |
|  | IL-8 | R=0.366;  **p=0.0002** | R=0.172;  p=0.087 | R=-0.092;  p=0.363 | R=0.138;  p=0.170 | R=0.150;  p=0.136 |
|  |  |  |  |  |  |  |

| **Sensitivity to dexamethasone^b^** | IL-1β | R=0.041;  p=0.661 | R=0.003;  p=0.975 | R=-0.012;  p=0.897 | R=-0.015;  p=0.868 | R=-0.015;  p=0.871 |
| --- | --- | --- | --- | --- | --- | --- |
|  | IL-6 | R=0.105;  p=0.316 | R=0.034;  p=0.744 | R=-0.073;  p=0.483 | R=0.055;  p=0.597 | R=-0.033;  p=0.754 |
|  | GM-CSF | R=0.116;  p=0.208 | R=0.140;  p=0.127 | R=-0.085;  p=0.358 | R=0.149;  p=0.105 | R=0.038;  p=0.684 |
|  | TNF-α | R=0.177;  p=0.056 | R=0.082;  p=0.380 | R=-0.124;  p=0.182 | R=0.121;  p=0.193 | R=-0.035;  p=0.708 |
|  | IL-8 | R=-0.055;  p=0.624 | R=-0.002;  p=0.988 | R=-0.106;  p=0.341 | R=0.136;  p=0.220 | R=-0.258;  **p=0.018** |

^a^ Measured as percentage of reduction in cytokine levels in LPS+dexamethasone-treated monocytes with respect to LPS-stimulated.

^b^ Measured as percentage of reduction in cytokine levels in LPS+dexamethasone-treated monocytes with respect to LPS-stimulated.

Correlations were performed using the Pearson’s correlation test.

Significant results are shown in bold.
