# Supplementary material for: Inflammatory dysregulation of monocytes in pediatric patients with obsessive-compulsive disorder
Source: J Neuroinflammation. 2017 Dec 28;14:261. doi: 10.1186/s12974-017-1042-z (PMC5746006; doi:10.1186/s12974-017-1042-z)
Supplement: Supplementary file 5 — Cytokine secretion by purified monocytes of early-onset OCD and healthy controls. (DOCX 24 kb) [file 12974_2017_1042_MOESM5_ESM.docx]

**Table S4.** Cytokine secretion by purified monocytes of early-onset OCD and healthy controls

|  | OCD patients | |  | Controls | | Statistic *F* | p-value |
| --- | --- | --- | --- | --- | --- | --- | --- |
|  | N | mean±SEM |  | N | mean±SEM |  |  |
| **Basal cytokine production (pg/ml)** | | | | | | | |
| IL-1β | 100 | 39.67 ± 4.32 |  | 43 | 38.21 ± 5.92 | 0.026 | 0.872 |
| IL-6 | 79 | 8.37 ± 1.14 |  | 39 | 14.34 ± 4.80 | 0.136 | 0.713 |
| GM-CSF | 100 | 1.46 ± 0.67 |  | 44 | 0.96 ± 0.26 | 0.954 | 0.330 |
| TNF-α | 98 | 12.86 ± 4.49 |  | 44 | 11.04 ± 4.81 | 0.073 | 0.787 |
| IL-8 | 79 | 2 357.07 ± 296.34 |  | 40 | 3,306.84 ± 803.93 | 0.010 | 0.920 |
| **Cytokine production after LPS stimulation (% of basal conditions)** | | | | | | | |
| IL-1β | 100 | 836.83 ± 61.55 |  | 43 | 550.10 ± 53.39 | 8.216 | **0.005**** |
| IL-6 | 79 | 57 377.43 ± 9 528.79 |  | 39 | 35 935.84 ± 8 456.03 | 8.570 | **0.004**** |
| GM-CSF | 100 | 20 505.34 ± 2 588.55 |  | 44 | 13 563.93 ± 2 762.77 | 4.433 | **0.049*** |
| TNF-α | 98 | 20 456.63 ± 2 391.81 |  | 44 | 14 274.75 ± 2 994.06 | 4.241 | **0.041*** |
| IL-8 | 79 | 2 390.30 ± 386.13 |  | 40 | 1 310.18 ± 245.87 | 6.330 | **0.013*** |
| **Cytokine production after LPS+dexamethasone treatment (% of basal conditions)** | | | | | | | |
| IL-1β | 100 | 458.23 ± 32.56 |  | 43 | 319.46 ± 27.03 | 5.528 | **0.020*** |
| IL-6 | 79 | 23 376.32 ± 4 975.85 |  | 39 | 16 287.88 ± 3 863.47 | 6.284 | **0.014*** |
| GM-CSF | 100 | 3 335.04 ± 434.83 |  | 44 | 2 218.67 ± 449.41 | 2.205 | 0.140 |
| TNF-α | 98 | 6 974.70 ± 955.62 |  | 44 | 4 956.34 ± 943.60 | 1.576 | 0.211 |
| IL-8 | 79 | 1 317.75 ± 173.89 |  | 40 | 673.31 ± 95.23 | 10.326 | **0.002**** |
| **Sensitivity to dexamethasone^a^** | | | | | | | |
| IL-1β | 100 | 40.76 ± 1.68 |  | 43 | 35.77 ± 2.98 | 1.332 | 0.251 |
| IL-6 | 79 | 49.14 ± 2.35 |  | 39 | 47.68 ± 3.40 | 0.609 | 0.437 |
| GM-CSF | 100 | 77.30 ± 1.99 |  | 44 | 71.07 ± 3.67 | 2.053 | 0.154 |
| TNF-α | 98 | 63.42 ± 1.89 |  | 44 | 53.37 ± 8.86 | 3.707 | 0.056 |
| IL-8 | 79 | 23.57 ± 7.39 |  | 40 | 32.54 ± 3.98 | 0.715 | 0.400 |

^a^ Measured as percentage of reduction in cytokine levels in LPS+dexamethasone-treated monocytes with respect to LPS-stimulated.

*p<0.05, **p<0.01. Significant results are shown in bold. Statistical analysis was performed using univariate general linear model adjusted for age and gender with natural-log-transformed data. Results expressed as means±SEM of the original data, prior to log-transformation.
