# Supplementary material for: Inflammatory dysregulation of monocytes in pediatric patients with obsessive-compulsive disorder
Source: J Neuroinflammation. 2017 Dec 28;14:261. doi: 10.1186/s12974-017-1042-z (PMC5746006; doi:10.1186/s12974-017-1042-z)
Supplement: Supplementary file 6 — Correlations of the duration of disease (expressed in months) and symptom severity (assessed by CY-BOCS score), with the percentage of total monocytes, monocyte subpopulations, and cytokine levels after LPS stimulation of monocytes in early-onset OCD. (DOCX 13 kb) [file 12974_2017_1042_MOESM6_ESM.docx]

**Table S5.** Correlations of the duration of disease (expressed in months) and symptom severity (assessed by CY-BOCS score), with the percentage of total monocytes, monocyte subpopulations and cytokine levels after LPS stimulation of monocytes in early-onset OCD.

|  | N | CY-BOCS score | | Disease duration |
| --- | --- | --- | --- | --- |
| **Total monocytes and monocyte subsets** | | |  | |
| Total monocytes (%) | 91 | R=-0.080; p=0.453 | | R=-0.079; p=0.454 |
| CD16+ monocytes^a^ | 91 | R=-0.178; p=0.091 | | R=0.048; p=0.653 |
| Classical monocytes^a^ | 91 | R=0.187; p=0.076 | | R=-0.067; p=0.530 |
| Intermediate monocytes^a^ | 91 | R=-0.125; p=0.237 | | R=-0.098; p=0.355 |
| Non-classical monocytes^a^ | 91 | R=-0.213; p=0.051 | | R=-0.067; p=0.530 |
| **Cytokine secretion after LPS stimulation (% of basal conditions)** | | | | |
| IL-1β | 100 | R=-0.010; p=0.921 | | R=-0.007; p=0.944 |
| IL-6 | 79 | R=-0.168; p=0.140 | | R=-0.122; p=0.284 |
| GM-CSF | 100 | R=-0.036; p=0.721 | | R=-0.062; p=0.538 |
| TNF-α | 98 | R=-0.114; p=0.265 | | R=0.017; p=0.866 |
| IL-8 | 79 | R=0.192; p=0.091 | | R=0.152; p=0.180 |

CY-BOCS: Children’s Yale-Brown Obsessive-Compulsive Scale

^a^ Expressed as percentage of total monocytes.

Correlations were performed using the Pearson's correlation test.
